# Out of pocket costs and time/productivity losses for pediatric sepsis in Uganda: a mixed-methods study

**DOI:** 10.1186/s12913-021-07272-9

**Published:** 2021-11-19

**Authors:** A. Krepiakevich, A. R. Khowaja, O. Kabajaasi, B. Nemetchek, J. M. Ansermino, N. Kissoon, N. K. Mugisha, M. Tayebwa, J. Kabakyenga, M. O. Wiens

**Affiliations:** 1 First Nations Health Authority, Vancouver, British Columbia Canada; 2 Faculty of Applied Health Sciences, Brock University, St. Catherines, Ontario Canada; 3 Walimu, Kampala, Uganda; 4 College of Nursing, University of Saskatchewan, Saskatoon, Saskatchewan Canada; 5 Department of Anesthesiology, Pharmacology and Therapeutics, University of British Columbia, Vancouver, BC Canada; 6 Center for International Child Health, BC Children’s Hospital, Vancouver, BC Canada; 7 Department of Pediatrics, University of British Columbia, Vancouver, Canada; 8 Mbarara University of Science and Technology, Mbarara, Uganda

## Abstract

**Background:**

Sepsis disproportionately affects children from socioeconomically disadvantaged families in low-resource settings, where care seeking may consume scarce family resources and lead to financial hardships. Those financial hardships may, in turn, contribute to late presentation or failure to seek care and result in high mortality during hospitalization and during the post discharge period, a period of increasingly recognized vulnerability. The purpose of this study is to explore the out-of-pocket costs related to sepsis hospitalizations and post-discharge care among children admitted with sepsis in Uganda.

**Methods:**

This mixed-methods study was comprised of focus group discussions (FGD) with caregivers of children admitted for sepsis, which then informed a quantitative cross-sectional household survey to measure out-of-pocket costs of sepsis care both during initial admission and during the post-discharge period. All participants were families of children enrolled in a concurrent sepsis study.

**Results:**

Three FGD with mothers (*n* = 20) and one FGD with fathers (*n* = 7) were conducted. Three primary themes that emerged included (1) financial losses, (2) time and productivity losses and (3) coping with costs. A subsequently developed cross-sectional survey was completed for 153 households of children discharged following admission for sepsis. The survey revealed a high cost of care for families attending both private and public facilities, although out-of-pocket cost were higher at private facilities. Half of those surveyed reported loss of income during hospitalization and a third sold household assets, most often livestock, to cover costs. Total mean out-of-pocket costs of hospital care and post-discharge care were 124.50 USD and 44.60 USD respectively for those seeking initial care at private facilities and 62.10 USD and 14.60 USD at public facilities, a high sum in a country with widespread poverty.

**Conclusions:**

This study reveals that families incur a substantial economic burden in accessing care for children with sepsis.

## Introduction

An estimated five million children worldwide die before their fifth birthday each year, primarily in sub-Saharan Africa and Southern Asia [1]. The Global Burden of Diseases project suggests that three million of these deaths can be attributed to sepsis [2]. The overwhelming burden of sepsis has also been outlined in the 2017 Resolution on Sepsis by the World Health Assembly, which recognizes sepsis as a public health issue of global concern [3]. The period immediately following discharge from the hospital is a significant period of vulnerability among children with sepsis. Growing evidence has shown that in many resource limited settings, as many children die following discharge as during admission, with most deaths occurring at home rather than during a subsequent readmission [4, 5]. Efforts to improve sepsis outcomes, therefore, must include a focus on the post-discharge period and work towards improving follow-up, especially among the most vulnerable, while also reducing barriers to accessing care for these families.

The economic burden attributable to pediatric sepsis in low-resource settings is very high. A recent study focusing on sub-Saharan Africa reported that neonatal sepsis is one of the most common reasons for admissions to intensive care units and represents a substantial economic burden to the health system [6]. It is conservatively estimated that 5.29–8.73 million disability-adjusted life-years (DALYs) are lost annually with an annual economic burden ranging from $10 billion to $469 billion. While the economic burden of sepsis on the health system may be recognized as critically important, there is little overall understanding of the costs absorbed by individual families for recurrent hospitalizations and post-discharge care of children with sepsis in Uganda. This is important since out of pocket (OOP) costs as well as time and productivity losses are the main drivers for timely care-seeking and choice of either public or private health facility. Furthermore, in countries like Uganda, where nearly half the population earns less than 1.90 USD/day [7], out of pocket costs could be catastrophic. Therefore, this study aimed to (1) explore a parental perspective of healthcare-seeking costs for children diagnosed with sepsis, and (2) estimate OOP costs, including time and productivity losses related to care-seeking practices representing both public and private not-for-profit (PNFP) health sectors in Uganda.

## Methodology

A mixed-methods study was conducted as part of a larger economic evaluation of the ongoing Smart Discharges study. Smart Discharges is a large multi-site study evaluating a discharge-focused intervention of counselling and a post-discharge follow-up referral among high-risk children to improve post-discharge survival. This study of 7000 children aged 6 to 60 months admitted with a proven or suspected infection is ongoing and will be completed in late 2021. The sites for evaluation include Mbarara Regional Referral Hospital (public), Holy Innocents Children’s Hospital (private), Masaka Regional Referral Hospital (public) and the Jinja Regional Referral Hospital (public). Children are followed up after discharge to determine post discharge mortality outcome.

This sub-study, embedded within the larger Smart Discharges study, collected data in two phases between October and December 2019. Phase I was a qualitative study comprised of focus group discussions (FGDs) with parents/primary caregivers of children admitted to the hospital for a severe infection, aiming to understand cost drivers related to pediatric sepsis care. Three FGDs with mothers and one with fathers were completed. A single paternal FGD was chosen, rather than three, as this was meant to add context and a paternal perspective to the maternal focus groups, and to aid in achieving thematic completeness. Phase II was a quantitative, 53-item, cross-sectional household survey designed to measure the OOP costs for pediatric sepsis care. The sampling frame for this study included parents/primary caregivers of children who had been enrolled in the Smart Discharges study and subsequently discharged from the hospital. Key variables in the survey instrument were guided by qualitative FGDs from Phase I. These included costs for ambulatory visits, medication, transport, and re-admission to a health facility during the first 2 months of post-discharge follow-up. In addition, data were collected on productivity/time losses of parents who missed work/wages due to the illness of their child.

Institutional ethics approval was obtained from the University of British Columbia (H19–02260), Mbarara University of Science and technology in Mbarara (21/8018) and the Uganda National Council of Science and technology (SS 4824).

### Study procedures

Phase I (Qualitative): One FGD was conducted in each of the three cities (Mbarara, Masaka, Jinja) by research assistants (RAs) who were trained to conduct the FGDs. All FGDs were conducted in the native languages of the three regions. A written informed consent was obtained from study participants prior to data collection. The FGD moderator guide was developed from previously published studies in LMICs and included open-ended questions related to OOP costs to parents and families including opportunity costs (productivity or time losses), health facility costs, post-discharge re-admission costs, and follow-up visit costs [8–10]. While one RA was moderating the FGD with questions and prompting questions, another RA was taking notes. A study manager was present to oversee the questions asked to members of the FGDs. A second team member reviewed transcripts prior to analysis.

Phase II (Quantitative): Key thematic areas pertinent to OOP costs and time/productivity losses identified in Phase I were included in the household survey. Identified variables included costs of ambulatory care, medication, transport, and re-admission. In addition, data were collected on productivity/time losses of parents who missed work/wages due to the illness of the child. The household survey was uploaded to REDCap, a secure web application for building and managing research data collection instruments [11]. This platform is specifically designed to support online or offline data capture for research studies. The REDCap platform runs on server infrastructure physically located at the BC Children’s Hospital Research Institute, in British Columbia, Canada. The survey employed multiple choice and short answer questions to better understand the cost values associated with these variables. Six RAs were trained to use e-tablets to complete the household survey. These RAs visited selected households and administered the survey questionnaire to mothers and family members of the child who had been discharged following an episode of sepsis. The RAs obtained written informed consent from all study participants prior to completing the survey. Follow-up phone calls were made for participants in some cases to resolve issues related to data entry errors or missing data.

### Sample size

For phase I, the desired number of FGDs were determined by data saturation [12]. Participants all had children enrolled in the Smart Discharges study.

For phase II, we estimated the minimum sample size using the standard formula (***n*** = [DEFF*Np (1-p)]/ [(d [2]**/Z** [2]_1-α/2_*(N-1) + p*(1-p)]) for cross-sectional studies. We used a population size of 7000 proven or suspected infections (on the sample size of the Smart Discharges study), assumed a rate of 90% for care-seeking in public or PNFP health sectors, a 5% margin of error and a design effect of 1.0 for random sampling. The design effect is widely used in survey sampling for planning a sample design and to report the effect of the sample design in estimation and analysis. This yielded a minimum sample size of 136 households. We inflated the resulting sample size by 10% to account for non-response, which translated into a final sample size of about 150 households. We randomly identified 50–55 households in each of the three cities (four study sites) from the Smart Discharges enrollment logs to partake in the household survey.

### Data analysis

Each FGD lasted about 1 hour, was audio-recorded, transcribed, and translated into English. Transcripts were analyzed using deductive content analysis, followed by inductive thematic analysis to generate the emerging themes/subthemes using Atlas ti.8 software [13]. For survey data, a descriptive analysis was performed to report on frequencies, proportions, means and standard deviations in SPSS software (IBM Statistics, Version 25). A t-test was applied to compare mean OOP costs for care seeking in the PNFP versus public sector health facilities and to calculate *p*-values.

## Results

### Phase 1: qualitative findings

Three FGDs were conducted with mothers/female caretakers (one in each study site). One FGD was conducted with fathers/male head of the household in Mbarara to understand and highlight any differing gender/role perspectives. A total of 27 out of 40 (67.5%) invited participants took part in four FGDs (~ 6–7 participants in each FGD) across the three study sites. Of 27 participants, 7 (25%) were male, and 20 (75%) were female. Caregivers ages ranged from 23 to 38 years, with a median age of 30.5 years. Findings are summarized based on three emerging themes: (i) financial losses, (ii) time and productivity losses, and (iii) coping with costs.

(i) Financial losses.

The OOP costs were financially burdensome, and adversely affected the overall well-being of all 27 study participants. Although public hospitals offer free services, almost all FGD participants reported having spent a significant amount of money regardless of whether the facility was PNFP or public. The most common reported expenses included paying for laboratory tests, radiology imaging services, and buying medications offered by private businesses outside the hospitals. Participants also frequently talked about the high cost of transportation to-and-from the hospital, meals for both child and the caregivers during admission, and airtime credit for phone calls. The total spending ranged between 5 and 550 USD, often dependant on the child’s condition and duration of hospitalization. Although services at public facilities are offered free of charge, some participants reported that nurses asked them to pay before their children could be seen by a care provider. One woman described the overall cost of visiting the health facility in the following quote:

“For transport, I paid 2,000 [shillings](~1USD) for the forward journey and another 2,000 [shillings](~1USD) for the return trip back home … and when we reached the clinic I paid 30,000 [shillings](~8USD) for treatment. For meals, I do not remember. When I took the child to Nyamitanga, I was charged 15,000 [shillings](~4USD) for transport, approximately 5,000 [shillings](~2USD) every day on meals and at discharge, I paid 372,000 [shillings](~100USD) for treatment. However, this cost had been subsidized by a certain organization, otherwise I would have ended up paying like 1,000,000 [shillings](~270USD)”. Participant R7, Women FG in Mbarara.

(ii) Time and productivity losses.

Almost all the FGD participants reported long-term financial effects as a result of their child’s primary stay in the hospital and/or re-admission. Parents/caregivers reported loss of daily wages, loss of employment, loss of assets such as crops, loss of capital, and death of animals due to the lengthy stay in hospital. In some cases, a temporary wage worker was hired to take care of other children or for household chores while the parent was at the hospital. These social and economic impacts were reported to have increased parents’/caregivers’ inability to care for their households. Six caregivers reported school dropout for other children in the family, four mentioned poor feeding, and eight participants reported loss of income or business. The remaining participants (*n* = 9) mentioned missing the planting seasons. Two caregivers explained their experiences in this way:

“The losses I incurred during treatment-seeking? By the time this child fell sick … I had procured a lorry of posho (maize flour). So I had to abandon the posho in the store, my husband is also a businessman in Kampala. I spent a month in Nyamitanga and by the time I returned home, the posho had already been infested with weevils [an insect] and the taste was sour... silence … I had paid 8,000,000 [shillings] (~2162USD) for a lorry of posho. So I ended up selling the posho cheaply to pig farmers”. Participant R2, Women FG in Mbarara.

I have three children and when one of them falls sick, it affects their feeding habits. For example, if I have been buying three cups of milk every day, it means that after the child has fallen sick, I will no longer be able to buy this milk because all the resources will be diverted to healthcare caregiving as well as clearing the debts incurred during the treatment seeking period.” Participant R7, FGD Men Mbarara.

#### Coping with costs

In order to pay for the expenses related to a child’s hospitalization, 23 (85%) of the FGD participants reported selling household items, including televisions, furniture, land, food, and livestock. 37% had borrowed money from their local village savings, 15% from friends, 11% from offices, 7% from banks, and 7% from family members, sometimes with daily or weekly interest. Half of the participants explained that their property such as land would be confiscated or would be reported to the local authorities if the borrowed money was not paid back on time. One of the caregivers detailed his experience with this quote:

“Sometimes parents sell off animals, others borrow from village saving groups because when the child falls sick or is already in hospital these savings groups act as a fallback position where one can borrow money and payback later though at a high interest rate. And sometimes because of failure to payback, people end up forfeiting their land. Sometimes children fail to go back to school because of the unforeseen expenses on medication”. Participant R2, Men FGD in Mbarara.

### Phase 2: quantitative findings

A total of 155 households were surveyed, of which 153 (99%) completed the questionnaire. Table 1 summarizes health resource utilization in children identified with suspected or proven infection during the three phases of observation: pre-hospitalization, hospitalisation and post-hospitalization. Overall, participants reported care seeking from 4 hospitals: 1 PNFP and 3 public. Eighty percent of children (*n* = 122) were admitted to Regional Referral Hospitals (RRH) for their primary hospitalization (public facilities) while the remaining 20% (*n* = 31), all from the Mbarara district, were admitted to the single PNFP hospital for their primary admission.

**Table 1:** Health care resource utilization of children identified with suspected or proven infection in the selected study sites

Variables	Private Sector	Public Sector (Regional Referral Hospital)			Overall ***N*** = 153
	Mbarara ***N*** = 31	Mbarara ***N*** = 19	Jinja ***N*** = 50	Masaka ***N*** = 53	
**A. Ambulatory visits prior to primary hospitalization**					
Visited health facility or care provider, n (%)	2 (6)	3 (16)	7 (14)	13 (25)	25 (16)
Common reasons for the visit, n (%)					
▪ Cough	2 (6)	–	–	3 (6)	5 (3)
▪ Diarrhea	–	–	1 (2)	2 (4)	3 (2)
▪ Fever	–	3 (16)	–	5 (9)	8 (5)
▪ Vomiting	–	–	1 (2)	–	1 (1)
▪ Other (Anemia, skin rash, oedema)	–	–	5 (10)	3 (6)	8 (5)
Type of health facility, n (%)					
▪ Public	–	2 (11)	6 (12)	11 (21)	19 (12)
▪ Private not for profit	2 (6)	1 (5)	1 (2)	2 (4)	6 (4)
Level of health facility, n (%)					
▪ Health Centre III	–	2 (11)	3 (6)	9 (17)	14 (10)
▪ Health Centre IV	–	–	3 (6)	3 (6)	6 (4)
▪ Clinic	2 (6)	1 (5)	1 (2)	1 (2)	5 (3)
**B. Primary hospitalization**					
Type of health facility, n (%)					
▪ Public	–	19 (100)	50 (100)	53 (100)	122 (80)
▪ Private not for profit	31 (100)	–	–	–	31 (20)
**Transport to- and from health facility (Multiple response: more than one option was applicable)**					
Mode of transport, n (%)					
▪ Public taxi / Boda-boda	27 (87)	17 (89)	47 (94)	40 (75)	131 (86)
▪ Personal vehicle / Boda-boda	1 (3)	1 (5)	–	6 (11)	8 (5)
▪ Neighbor’s vehicle	–	–	–	1 (2)	1 (1)
▪ Hospital ambulance	1 (3)	–	–	–	1 (1)
▪ Other (motorcycle, relative’s vehicle, special hire)	2 (6)	1 (5)	3 (6)	6 (11)	12 (8)
**Meals purchased during hospitalization (Multiple response: more than one option was applicable)**					
Persons purchased meals, n (%)					
▪ Child hospitalized	22 (71)	15 (79)	45 (90)	40 (75)	122 (80)
▪ Accompanying parents or family member	27 (87)	19 (100)	48 (96)	48 (91)	142 (93)
▪ Other (sibling of hospitalized child, neighbor)	13 (42)	8 (42)	3 (6)	2 (4)	26 (17)
**Productivity losses due to hospitalization of the child (Multiple response: more than one option was applicable)**					
Persons missed daily wages, n (%)					
▪ Mother only	19 (61)	11 (58)	23 (46)	21 (40)	74 (48)
▪ Father only	–	1 (5)	10 (20)	1 (2)	12 (8)
▪ Both parents	11 (35)	7 (37)	9 (18)	–	27 (18)
▪ Other family member / relative	1 (3)	–	5 (10)	–	6 (4)
Occupation of persons who missed wages, n (%)					
▪ Skilled worker	–	–	2 (4)	9 (17)	11 (7)
▪ Non-skilled worker	1 (3)	–	1 (2)	1 (2)	3 (2)
▪ Farming or agriculture	6 (19)	6 (32)	19 (38)	5 (32)	36 (24)
▪ Private business	13 (42)	7 (37)	17 (34)	10 (37)	47 (31)
▪ Employed	6 (19)	–	5 (10)	–	11 (7)
▪ More than one occupation	5 (16)	6 (32)	4 (8)	6 (11)	21 (14)
Hired someone on daily wages to take care of child or complete household chores, n (%)	13 (42)	11 (58)	1 (2)	1 (2)	26 (17)
**C. Post-discharge referral and follow-up care**					
Child referred to lower level health facility for follow-up care, n (%)	24 (77)	18 (95)	34 (68)	27 (51)	103 (67)
Children sought follow-up care, n (%)	20 (65)	16 (84)	30 (60)	22 (42)	88 (58)
Type of health facility, n (%)					
▪ Public	7 (23)	13 (68)	25 (50)	22 (42)	67 (44)
▪ Private not for profit	13 (42)	3 (16)	5 (10)	–	21 (14)
Level of health facility or care provider, n (%)					
▪ Facility health worker	20 (65)	16 (84)	27 (54)	18 (34)	81 (53)
▪ VHT	–	–	2 (4)	4 (8)	6 (4)
▪ Other	–	–	1 (2)	–	1 (1)
Child readmitted to a health facility, n (%)	5 (16)	6 (32)	12 (24)	7 (13)	30 (20)
Completed laboratory tests or imaging, n (%)	12 (39)	7 (37)	1 (2)	1 (2)	21 (14)
Purchased medical supplies or equipment, n (%)	1 (3)	1 (5)	5 (10)	1 (2)	8 (5)
Purchased medications, n (%)	10 (32)	5 (26)	12 (24)	11 (21)	38 (25)
**D. Financial burden to families**					
Had to sell household assets, n (%)	14 (45)	15 (79)	9 (18)	10 (19)	48 (31)
Types of assets sold					
▪ Electronics	2 (6)	2 (11)	2 (4)	2 (4)	8 (5)
▪ Furniture	1 (3)	1 (5)	–	–	2 (1.3)
▪ Land	–	1 (5)	1 (2)	–	2 (1.3)
▪ Animals	11 (35)	8 (42)	4 (8)	5 (9)	28 (18)
▪ Birds	–	2 (11)	6 (12)	2 (4)	10 (7)
▪ Other (food, utensils, business stocks)	1 (3)	3 (16)	5 (10)	1 (2)	10 (7)

**Fig. 1:**
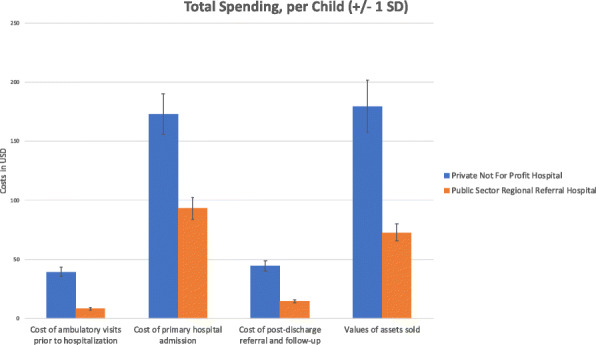
Summary of the total spending per child

As expected, the average OOP costs for the primary hospital admission fee (Table 2B) was higher at the PNFP site than the three public sites (Fig. 1), with the PNFP averaging 115,290 Ugandan Shillings (UGX) (~ 31.20 USD) per admission and the public health sector averaging 10,656 UGX (~ 2.88 USD) per admission (*p*-value: 0.005). For context, in 2015 about 40% of Ugandans earn less than 1.90 USD/day [20]. Differences were also noted between public sector facilities, though these were smaller than those found between PNFP and public institution (Table 2). Similar to what was observed with admission-related costs between PNFP and public facilities, there was also a significant difference in the average spending on medications, with average OOP costs approximately 27 USD compared to 11 USD between PNFP and public facilities, respectively (*p*-value of < 0.0001). Total mean costs for hospital admission, excluding missed wages, were 124.50 USD for those seeking care at private facilities, compared to 62.10 USD for those seeking care at public facilities.

**Table 2:** Out-of-pocket costs and time/productivity losses

Costs, in USD	Private Not For Profit Hospital (***N*** = 31)		Public Sector Regional Referral Hospital (***N*** = 122)		***p***-value
	Mean (USD)	Std. d	Mean (USD)	Std. d	
**A. Costs of ambulatory visits prior to hospitalization, per child**					
Fee for service	39.4	40.4	8.3	10.9	0.005
**B. Costs related to primary hospitalization, per child**					
Hospital fee	31.4	17.4	2.9	9.8	< 0.0001
Airtime and other forms of communication	2.1	1.4	2.4	1.8	0.373
Medications	27.6	15.6	11.2	17.2	< 0.0001
Transport to- and from health facility	32.1	25.2	17.3	24.2	0.003
Meals for the sick child and caretakers	23.1	22.9	16.9	26.9	0.240
Missed wages by parents and family members	48.6	87.1	31.2	67.6	0.233
Hired paid worker to take care of child	8.2	6.0	11.4	13.9	0.407
**Total hospitalization cost, excluding missed wages (USD)**	124.5		62.1		–
**C. Costs of post-discharge referral and follow-up care, per child**					
Follow-up ambulatory visits	4.9	7.6	1.9	7.6	0.123
Re-admissions	30.8	32.7	10.7	17.9	0.057
Laboratory tests	3.5	8.5	0.2	0.9	0.002
Medications	5.3	6.4	1.6	4.5	0.004
Medical equipment or supplies	0.1	0.5	0.2	0.7	0.602
**Total post-discharge cost (USD)**	44.6		14.6		–
**D. Financial risks to family due to hospitalization and/or ambulatory care of the child, per child**					
Value of assets sold	179.5	224.5	72.8	85.1	0.020

Over 80% (*n* = 131) of caregivers reported using a public taxi / Boda-boda (motorcycle taxi) to travel to the health facility (Table 1B) with a median of 6 trips [IQR: 4–10], with a reported average cost of 17 USD for these trips compared to 32 USD in PNFP (*p*-value of 0.003) (Table 2B). In most cases (*n* = 74 out of 83; 89%), caregivers who were working in the farming/agriculture sector or private business missed work wages (median 4 days [IQR: 0–7], and an average 31 USD), largely reflecting the same prominent thematic element noted in FGDs. Most caregivers were required to purchase meals either for themselves (*n* = 142, 93%) or for their admitted child (*n* = 122, 80%), with an average OOP cost of 17 USD. Communication (airtime), a common need identified in the FGDs, resulted in an average cost of approximately 2 USD for those admitted to either a PNFP or public facility (*p*-value of 0.373). Participants (especially men in Mbarara) spent between 1 and 17 USD on airtime to make phone calls. The airtime was mainly used to update family members on the child’s status, mobilise transport and food. Costs related to maintaining the household while the caregiver was away was also identified most often in the Mbarara region (24 of 50 participants, 48%), but less in Masaka or Jinja (2 of 103 participants, 2%).

Approximately 16% (*n* = 25) of parents sought ambulatory care from a health facility or care provider prior to a reported re-admission (Table 1C), with an average cost of 39 USD per child (taking into account multiple visits before readmission) in the PNFP sector compared to 8 USD in the public sector (*p*-value of 0.005) (Table 2C). Of thirty children who required re-admissions, 15 (50%) went to public sector hospitals. The average cost of re-admission was 11 USD in the public sector hospital, in contrast to the much higher cost (31USD) in the PNFP sector. Overall, costs were higher in the Mbarara district, likely due to this district having the only PNFP hospital involved in this study.

As a part of their participation in the Smart Discharges study, 103 children (67%) involved in this study were deemed to be at a high risk of post-discharge mortality and were thus referred to the lower-level health facilities for follow-up care (Table 1C). Of these, 88 children (85%) completed at least one follow-up visit, with 67 (44%) completing at least 2 visits. The follow-up visit was costly (5 USD) for those seeking care at a private facility, whereas it was relatively less costly (2 USD) for those seeking follow-up care at a public facility / care provider (Table 2C).

Overall, as a means of generating additional income to cover necessary health expenses related to the care of their child, one-third of parents reported selling household assets (Table 1D). The average value of these assets ranged from 73 USD to 180 USD, for those who were initially admitted to a public vs PNFP facility, respectively (Table 2D). Most commonly sold assets include animals (*n* = 28, 18%), birds (*n* = 10, 7%), and electronics (*n* = 8, 5%).

## Discussion

To our knowledge, this is the first study providing important context around the short-term and potentially long-term financial burden of pediatric sepsis on families, including the costs related to the period immediately following discharge in Uganda. This study revealed that expenses are high during the initial admission, subsequent readmissions, and follow-up care, with the primary admission incurring the highest cost. Substantial costs were incurred in both public or private systems, although these were higher in private facilities. Costs were primarily observed to be related to transport to and from the facility, medication, airtime, meals, and missed wages as well as the admission cost at the PNFP hospital. A child’s illness has the potential to create a further economic burden related to loss of assets such as land and livestock, which are vital to ensure the future wellbeing of the family.

Our results suggest that OOP costs incurred both during an admission and in the post-discharge period have the ability to push households into poverty and discourage return for future care. Aregbeshola and colleagues aggregated data from multiple studies across Africa and found that OOP costs for healthcare often resulted in the impoverishment of families [14]. In Uganda specifically it was found that these costs led to an annual relative poverty rate increase of 18.1%, and absolute poverty rate increase of 4.1% [14, 15]. Data from both Nigeria and Vietnam found that families who were more impoverished were overall more likely to seek lower levels of care, self-medicate, or not seek care at all due to the extent of the financial burden it places on already impoverished families [14, 16]. Overwhelmingly it is found that poor and marginalized families seek healthcare less frequently or even forgo healthcare more often than those of a higher socioeconomic status due to expense or inaccessibility [17, 18].

A devastating impact of seeking care was the need to sell assets to raise necessary funds, a practice called distress financing. Other burdens included loss of employment and property, such as the death of animals while attending to the sick child. The downstream impact of loss of earnings and property assets may stultify children’s long-term prospects when they are forced to drop out of school due to the inability of the family to pay school fees. Seeking treatment from an herbalist/healer or buying drugs recommended by staff at drug-shops and pharmacies rather than seeking treatment at a facility was often expressed by participants in the FDGs; the sum total of these may lead to avoidance of or distrust in the health care system [18, 19].

There was a substantial difference across most domains in the cost for primary admission between private and public institutions. Though private care is more widely desired by Ugandans largely due a perception of better quality, costs are prohibitive to most. Among caretakers who missed wages, with typical losses of between 8000 to 12,000 UGX (about 2–3 USD) per day while their child was admitted to the hospital. This, along with the costs of care, are tremendous in a country where the per-capita GDP is 817 USD and where 40% of Ugandans earn less than 1.90 USD/day [7, 20]. Thus, the financial costs and the impact of missed wages often have enormous impact on the family of a child with sepsis.

The results of our study emphasize the effects of OOP health expenditure on families of children with infectious disease in arguably the least-impoverished regions of Uganda (central and southwest), limiting our findings geographically; however, this study as well as others done in LMICs, suggest that the issues identified by this research may be even greater in regions already suffering enormous levels of poverty. However, more research is required to fully understand the extent of economic impact on families both in the more impoverished regions of Uganda as well as within Kampala as the capital city, where costs are likely to differ. The sample size for both the FGDs and the household survey were moderate, which serves as another limitation. Nevertheless, the high survey response rate and detailed qualitative analysis are integral in guiding future efforts to understand and characterise costs in other areas of Uganda. Finally, cost reporting bias may have also been present due to poor/differential recall according to illness severity, which parent was interviewed (mother/father), as well as the time elapsed since the events occurred. These are limitations with all retrospective data collection and justify the need for prospective economic research on sepsis OOP costs in the future.

## Conclusion

This study revealed the substantial economic burden placed on families who deal with sepsis-related pediatric care in Uganda. Findings highlighted significant cost disparities between the public and private healthcare sectors, although also emphasizes the consistent barrier that cost can be to accessing health care for children regardless of private or public facility. This study provides important cost parameters for future economic studies, including the currently ongoing Smart Discharges program’s cost-effectiveness analysis.

## Data Availability

Datasets used in the current study are available from the corresponding author on reasonable request.

## Declarations

Ethics approval and consent to participate: Institutional ethics approval was obtained from the ethics committee at the Office of Research Ethics at University of British Columbia (H19–02260), the Research Ethics Committee at Mbarara University of Science and technology in Mbarara (21/8018) and the Uganda National Council of Science and technology (SS 4824). All methods used in this research were in accordance with relevant guidelines and regulations. Informed consent was obtained from all subjects and/or their legal guardian(s).
